# Seroprevalence and risk factors for *Toxoplasmosis* in HIV infected and non-infected individuals in Bahir Dar, Northwest Ethiopia

**DOI:** 10.1186/1756-3305-6-15

**Published:** 2013-01-16

**Authors:** Fisseha Walle, Nigatu Kebede, Aster Tsegaye, Tesfu Kassa

**Affiliations:** 1 Department of Immunology and Hematology, Bahir Dar Regional Health Research Laboratory, Bahir Dar, Ethiopia; 2 Aklilu Lemma Institute of Pathobiology, Addis Ababa University, Addis Ababa, Ethiopia; 3 Department of Pathology, College of Medicine, Ohio State University, Columbus, OH 43210, USA; 4 Department of Medical Laboratory Sciences, Addis Ababa University, Addis Ababa, Ethiopia

**Keywords:** *Toxoplasmosis*, *Toxoplasma gondii*, IgG, IgM, HIV, Seroprevalence

## Abstract

**Background:**

*Toxoplasmosis*, a zoonotic disease distributed worldwide, is an infection caused by the ubiquitous obligatory intracellular coccidian protozoan organism, *Toxoplasma gondii*. It is a major public health concern because the disease is serious in terms of mortality or physical and /or psychological sequellae in patients with HIV disease. The aim of the study was to assess the seroprevalence of *Toxoplasma gondii* IgG and IgM antibodies and associated risk factors in HIV infected and non-infected individuals attending Felege Hiwot referral hospital, Bahir Dar, Northwest Ethiopia.

**Methods:**

A cross sectional study was conducted at Felege Hiwot referral hospital, Bahir Dar, Amhara National Regional State. Venous blood samples were collected from 103 HIV infected pre anti-retroviral therapy patients at Felege Hiwot referral hospital and 101 HIV negative apparently healthy voluntary blood donors at the blood bank. Serum samples were analyzed for anti-*Toxoplasma gondii* IgG and IgM antibodies using a commercially available ELISA kit. Socio-demographic and associated risk factors for *Toxoplasmosis* from each individual were also obtained and the data was analyzed using SPSS version 18.

**Results:**

Of the examined HIV seropositive individuals, 87.4% (90/103) and 10.7% (11/103) were positive for anti-*T*. *gondii* IgG and IgM antibodies, respectively. Multivariate analysis using logistic regression showed that anti-*T. gondii* seropositivity was independently significantly associated with undercooked or raw meat consumption (adjusted OR=5.73, 95% CI=1.35-24.39; P=0.02) and having contact with cat (adjusted OR= 4.29, 95% CI=1.08-16.94; P=0.04) in HIV positive individuals. In HIV negative apparently healthy blood donors, prevalence of anti-*T. gondii* antibodies were 70.29% and 2.97% for IgG and IgM, respectively. Multivariate analysis showed that undercooked or raw meat consumption (adjusted OR=6.45, 95% CI=2.16-19.28; p=0.001) and sex (OR=6.79, 95% CI=2.14-21.60; p=0.001) were independently significantly associated with anti-*T. gondii* IgG seropositivity, with a significantly higher number of males affected than females.

**Conclusion:**

The present findings showed a high sero-prevalence of anti-*T. gondii* antibodies in HIV infected pre-ART and HIV non-infected apparently healthy blood donors in Bahir Dar. Consumption of undercooked or raw meat might greatly contribute towards acquiring *T. gondii* infection in HIV infected pre-ART and HIV non-infected apparently healthy blood donors. It may be appropriate to include routine serological screening test for determination of anti-*T. gondii* antibodies in HIV infected pre-ART individuals and HIV negative apparently healthy blood donors. In addition, health education towards avoiding eating undercooked and raw meat, and avoiding contact with cats were recommended.

## Background

Toxoplasmosis is a globally distributed zoonosis [1] caused by the ubiquitous obligatory intracellular coccidian protozoan organism, *Toxoplasma gondii* that infects a wide range of animals, man and birds [2,3]. *T. gondii* is an opportunistic parasitic infection in immune compromised hosts [4,5] and estimates indicate that up to one third of the world's human population is infected [6]. Prevalence of the infection varies widely, depending on social and cultural habits, geographic factors, climate, and transmission route. It has been reported that the prevalence is higher in warm and humid areas [7]. The parasite causes chronic infection in adults and is present in an estimated 22.5% of people older than 12 in the United States [8] and up to 90% of the population in other regions of the world [9].

*T. gondii* is transmitted to humans by eating raw or inadequately cooked infected meat [10]; through ingestion of oocysts that cats have passed in their feces and women can transmit the infection transplacentally to their unborn fetus. Other infection pathways are transfusion, transplantation and direct contamination [11]. In adults, the incubation period for *T. gondii* infection ranges from 10 to 23 days [12].

Toxoplasmosis is a major public health concern because the disease is serious in terms of mortality or physical and /or psychological sequellae in patients with HIV disease [13]. In the majority of normal, healthy (immune competent) subjects, infection is asymptomatic [7,14] and frequently results in the chronic persistence of cysts within host tissues; the cysts normally lie dormant, probably for life [14]. But, in immune compromised states such as in HIV infections, subjects are at risk of developing acute toxoplasmosis due to reactivation of the organism if their CD4+ T-cell count decreases below 200 cells/μL [15,16]. Since the pandemic of HIV infection has spread throughout the world, toxoplasmosis has been implicated as one of the most important opportunistic infections in HIV/AIDS patients [17-19]. Moreover, in up to 10% of HIV infected immune competent individuals, it causes cervical lymphadenopathy or ocular disease [19].

In the immunocompromised patients, diseases due to *T. gondii* are generally considered to represent reactivation of latent infection [20]. Detection and monitoring of anti-*Toxoplasma* antibodies are of a great interest in HIV-infected patients. Ethiopia is one of the high HIV epidemic countries [21] with approximately 1.5 million people infected and living with HIV/AIDS. About 5-7% of patients live in and around Bahir Dar and its neighboring areas. However, the *Toxoplasmosis* serologic status and risk factors of infection among HIV-infected patients and HIV non-infected apparently healthy individuals remain unknown. Therefore, this study aimed to determine the seroprevalence of anti-*T. gondii* IgG and IgM antibodies in HIV infected and non-infected individuals and to identify the possible risk factors associated with toxoplasmosis in Bahir Dar, northwest Ethiopia.

## Methods

### Study area and study subjects

A cross sectional study was conducted to determine the seroprevalence and risk factors of toxoplasmosis in HIV infected and non-infected individuals at Felege Hiwot referral hospital, Bahir Dar, Amhara National Regional State, northwest Ethiopia. Bahir Dar is the capital of Amhara National Regional state, located approximately 578 km Northwest of Addis Ababa, having a latitude and longitude of 11°36^′^N 37°23^′^E11.6°N 37.383°E11.6; 37.383 coordinates and an elevation of 1840 meters above sea level. Based on the 2007 Census conducted by the Central Statistical Agency of Ethiopia (CSA), this city has a total human population of 221,991 [22].

The study participants were individuals who visited the hospital to know their HIV sero-status and to donate blood for their families. Thus, the study subjects were HIV sero-positive pre-ART individuals attending the pre-ART clinic of Felege Hiwot referral hospital (Group1), and HIV sero-negative apparently healthy adults who came to the hospital for blood donation (Group 2). The convenient sampling method was followed.

Inclusion criteria of the study subjects in Group 1 were: 1) HIV sero-positive ART naive individuals attending the pre-ART clinic for further management; 2) aged 18 years and older; 3) not taking chemoprophylaxis for toxoplasmosis; and in Group 2 were: 1) voluntary blood donors (HIV, HCV, HBs Ag and *Treponema pallidum* sero-negatives); 2) aged 18–55 years; in both cases individuals who accepted to participate in the study were considered.

### Data collection

Five milliliters of venous blood without anticoagulant was aseptically collected from 103 HIV positive ART naive and 101 HIV negative apparently healthy voluntary blood donors at Felege Hiwot referral hospital pre-ART and blood bank laboratories, respectively. Collected blood samples were transported immediately to Bahir Dar Regional Health Research Laboratory. In the laboratory serum was separated, labeled and stored at −20°C until use.

Using a pretested questionnaire, data on socio-demographic characteristics and associated risk factors were collected from all study participants.

### Serological assays

Serum samples were screened for anti-*T. gondii* IgG and IgM antibodies by ELISA using "Toxoplasma IgG and IGM" kit (Human, GmbH.65205 Wiesbaden-Biochemical Diagnostic, Germany) according to the manufacturers’ instructions. Positive and negative controls were used with each series of anti *T. gondii* IgG/IgM test (Human, Germany); results were obtained by comparison with a cut-off value measured at 450nm absorbance.

### Ethical approval

Ethical clearance to conduct the study was obtained from Aklilu Lemma Institute of Pathobiology (ALIPB), Addis Ababa University (AAU) and Amhara National Regional State Health Bureau (ANRSHB). The purpose and procedures of the study were explained and a written informed consent was obtained from all study participants. Study participants with positive test result for toxoplasmosis were treated following standard protocols at Felege Hiwot referral hospital, following the FMOH (Federal Ministry of Health), guidelines [23].

### Statistical analysis

Data was entered into MS Excel, cleaned and analyzed using SPSS software, with Version 18.0. Pearson Chi-square, Odds Ratio and 95% confidence interval were used for group comparison and association of variables. *P* values were determined and taken as a level of significance when found less than 0.05.

## Results

The overall prevalence of anti-*T. gondii* IgG antibodies in HIV positives pre-ART individuals and apparently healthy blood donors were found to be 87.4% (90/103) and 70.29% (71/101), respectively. The difference between the two groups in anti-*T. gondii* IgG seropositivity was statistically significant (p=0.003). Similarly, the overall anti-*T. gondii* IgM seropositivity was 10% (11/103) among HIV infected Pre ART and 2.97 % (3/101) in HIV non-infected study participants; the difference was statistically significant (p=0.02). Overall, the prevalence of anti-*T. gondii* antibodies in HIV positives pre-ART individuals was significantly higher than HIV non-infected apparently healthy blood donors (Tables 1, 2).

**Table 1:** **Seroprevalence of *Toxoplasma *infection in HIV infected pre-ART individuals according to socio demographic and other risk factors**

**Variable**	**Frequency (%)**	**IgG +ve (%)**	P value^*^	**IgM +ve(%)**	**P *value***
**Age in years**			0.45		0.78
18-20	3(2.9)	3(100)		0 (0.0)	
21-30	43(41.8)	35 (81.4)		4(9.3)	
31-40	31(30.1)	28(90.3)		3(9.7)	
41-55	26(25.2)	24(92.3)		4(15.4)	
**Sex**			0.39		0.70
M	43(41. 7)	39 (90.7)		4 (9.3)	
F	60 (58.3)	51(85.0)		7 (11.7)	
**Place of residence**			0.29		0.75
Urban	96(93.2)	83(86.5)		10(10.4)	
Rural	7(6.8 )	7(100)		1(14.3)	
**Education**			0.20		0.68
Literate	71(68.9)	64(90.1)		7(9.9)	
Illiterate	32(31.1)	26(81.3)		4(12.5)	
**History of blood transfusion**			0.27		0.19
Yes	3(2.9)	2(66.7)		1 (33.3)	
No	100(97.1)	88(88.0)		10(10.0)	
**Contact with cat**			0.04		0.83
Yes	72(69.9)	66(91.7)		8(11.1)	
No	31(30.1)	24 (77.4)		3(9.7)	
**Eating undercooked or raw meat**			0.02		0.31
Yes	86(83.5)	78(90.7)		8(9.3)	
No	17(16.5)	12(70.6)		3(17.7)	
**Eating unwashed raw vegetables or fruits**			0.85		0.82
Yes	50(48.5)	44(88.0)		6(12.0)	
No	53(51.5)	46(86.8)		5(9.4)	
**Total**	103(100)	90(87.4)		11 (10.7)	

*P values were calculated using Chi square test.

**Table 2:** **Seroprevalence of *Toxoplasma *infection in HIV non-infected apparently healthy blood donors according to socio demographic and other risk factors**

**Variable**	**Frequency (%)**	**IgG +ve(%)**	P value^*^	**IgM +ve(%)**	**P *value***
**Age in years**			0.06		0.52
18-20	29 (28.7)	15(51.7)		1(3.5)	
21-30	53(52.5)	41(77.4)		1(1. 9)	
31-40	9(8.9)	8(88.9)		0 (0.0)	
41-55	10(9.9)	7(70.0)		1 (10.0)	
**Sex**			0.001		0.54
M	50(49.5)	44(88.0)		2 (4.0)	
F	51(50.5)	27(52.9)		1 (2.0)	
**Place of residence**			0.88		0.25
Urban	67(66.3)	46 (68.7)		1 (1.5)	
Rural	34(33.7)	25 (73.5)		2 (5.9)	
**Education**			0.86		0.23
Literate	66(65.4)	46(69.7)		1 (1.5)	
Illiterate	35(34.7)	25(71.4)		2(5.7)	
**History of blood transfusion**					
Yes	0	0		0(0.0)	
No	101(100)	71(70.3)		3(3.0)	
**Contact with cat**			0.81		0.95
Yes	69(68.3)	48(69.6)		2 (2.9)	
No	32(31.7)	23(71.9)		1 (3.1)	
**Eating undercooked or raw meat**			0.001		0.17
Yes	63 (62.4)	53 (84.1)		3 (4.8)	
No	38 (37.6)	18 (47.4)		0 (0.0)	
**Eating unwashed raw vegetables or fruits**			0.17		0.82
Yes	61(60.4)	46(75.4)		2(3.3)	
No	40(39.6)	25(62.5)		1(2.5)	
Total	101(100%)	71(70.29%)		3/101(2.97)	

*P values were calculated using Chi square test.

In HIV infected pre-ART individuals studied, where the majority reported having contact with cats 72/103 (69.9%) and the habit of eating raw or undercooked meat 86/103 (83.5%), the bivariate analysis showed a significant association between these factors and seropositivity to anti-*T. gondii* IgG antibodies (Table 1). No significant association was observed between seropositivity for anti-*T. Gondii* IgG antibodies and educational status, eating unwashed raw vegetables or fruits, residence (urban versus rural inhabitants). In addition, the prevalence of anti-*T. gondii* IgG, among the HIV positive pre-ART individuals was not statistically influenced by the sex and age categories tested. In the study there were only three individuals with history of blood transfusion, and all of them were HIV positive.

In HIV infected pre-ART individuals, no statistically significant association was detected between anti-*T. gondii* IgM seropositivity and all the risk factors evaluated.

In HIV non-infected apparently healthy voluntary blood donors, among the demographic and other risk factors studied, consumption of undercooked or raw meat and sex of the individual was significantly associated with anti-*T. gondii* IgG seropositivity. The rate of anti-*T. gondii* IgG seropositivity was significantly higher in males than females, and in those eating raw or undercooked meat compared to those who did not (Table 2).

As shown in Table 3, multivariate analysis using logistic regression showed that undercooked or raw meat consumption (adjusted OR=5.73, 95% CI=1.35-24.39, p=0.02) and having contact with cat (adjusted OR= 4.29, 95% CI=1.08-16.94, p=0.04) were independently associated with anti *Toxoplasma* IgG seropositivity in HIV infected patients. Whereas in HIV uninfected participants, undercooked or raw meat consumption (adjusted OR=6.45, 95% CI=2.16-19.28; p=0.001) and being male (adjusted OR=6.79, 95% CI=2.14-21.60, p=0.001) were independently significantly associated with anti-*T. gondii* IgG seropositivity (Table 4). In addition, being in the age group 21–30 was significantly associated with *T. gondii* IgG seropositivity compared to the reference age group 18–20 years (adjusted OR 5.58, 95% CI=1.62-19.29, p=0.01).

**Table 3:** **Multivariate analysis of selected characteristics of 103 HIV infected Pre-ART individuals and their association with anti-IgG to *T. gondii *infection**

**Characteristics**^**a**^	**Adjusted odds ratio**^**b**^	**95% confidence interval**	**P value**
**Education**			
Literate	1.31	0.35-4.87	0.69
Illiterate	1		
**Contact with Cat**			
Yes	4.29	1.08-16.94	0.04
No	1		
**Eating under cooked or raw meat**			
Yes	5.73	1.35-24.39	0.02
No	1		

^a^ The variables included were those with P≤0.2 obtained in the bivariate analysis.

^b^ Odds ratio adjusted for variables included in the table.

**Table 4:** **Multivariate analysis of selected characteristics of 101HIVnegative apparently healthy voluntary blood donors and their association with anti-IgG to *T. gondii *infection**

**Characteristics**^**a**^	**Adjusted odds ratio**^**b**^	**95% confidence interval**	**P value**
**Sex**			
Male	6.79	2.14-21.60	0.001
Female	1		
**Age**			
18-20	1	1.62-19.29	0.01
21–30	5.58	0.73-104.47	0.09
31–40	8.71	0.31-14.04	0.45
41-55	2.09		
**Eating under cooked or raw meat**			
Yes	6.45	2.16-19.28	0.001
No	1		

^a^ The variables included were those with P≤0.2 obtained in the bivariate analysis.

^b^ Odds ratio adjusted for variables included in the table.

## Discussion

Toxoplasmosis refers to the disease caused by *T. gondii*[24]. In the present study *Toxoplasma* specific IgG and IgM antibody levels were analyzed using ELISA, one of the standard procedures for detection of antibodies [18]. Detection of both IgG and IgM simultaneously helps establishing exposure to *T. gondii* and the chronological status of such exposure. In the present study the prevalence rate of anti-*T. gondii* IgG antibodies in HIV positive pre-ART individuals was significantly higher than in HIV negative blood donors (87.4% versus 70.29%, P=0.003). Similar finding of significantly higher *Toxoplasma* seropositivity in HIV infected individuals than in HIV-uninfected people was also demonstrated in Addis Ababa [25]. The finding of significant difference between HIV infected and non-infected individuals also agrees with studies done in Bamako, 60% versus 21% [26] and in Nigeria 54% versus 37.5% [27]. These differences might be attributed to the reactivation of latent infection/tissue toxoplasmosis when the host immunity is compromised (reduced cellular immunity) in HIV patients [28]. In addition, variability in results can be due to differences in age among the study groups and differing lifestyles and geographical conditions. In our case, HIV infected patients were older compared to the HIV negative counterparts (median age 23 versus 34 years, p=0.000).

The overall finding of a high seroprevalence of IgG in HIV positive pre-ART individuals was comparable with the study done in Akaki town, suburban of Addis Ababa, in which sera were screened for anti-*Toxoplasma* IgG antibodies using the Sabin-Feldman test and 80% seroprevalence was reported [29]. Our finding is also consistent with a Venezuelan study which demonstrated 85% prevalence of anti-*T. gondii* IgG antibody in the HIV-positive adult population [15]. However, our result was much higher than other studies as in Johannesburg 8% [30]. It also differs from a study done in Iran among HIV/AIDs patients which reported prevalence of 58% [31]. The observed differences in prevalence of anti-*T. gondii* antibodies could be due to differences in geographical distribution and/ or possible risk factors and socioeconomic conditions [32] contributing to acquiring the infection.

In the present study, anti-*T. gondii* IgM seropositivity rate was 10.7% in HIV infected pre-ART individuals. A similar study in India reported 6% anti-*Toxoplasma* IgM positivity using double sandwich ELISA [14]. The consumption of undercooked or raw meat and contact with cat were found to be risk factors for the presence of anti-*T. gondii* IgG in HIV infected pre-ART individuals. Similar results were demonstrated by studies done in Nazaret town, Ethiopia [3] and at Lagos University teaching hospital, Nigeria [27]. On the other hand, no significant association was identified between the history of blood transfusion and anti-*T. gondii* seropositivity. It is worth mentioning that the number of participants with a history of blood transfusion was only three and all of them were HIV positive. Nonetheless, a similar study in Malaysia has documented the absence of a significant association between a history of blood transfusion and *T. gondii* seropositivity [24]. Beef, lamb and chicken are eaten in the study area. In the nation raw meat dishes such as "Kitfo" and "kourt" are most favored by the society and eaten raw or lightly cooked. However, HIV positive individuals are advised to eat cooked meat to minimize persistent immune activation as a result of tapeworms and or other secondary infections through meat as immune activation is one of the mechanism of CD4 depletion. HIV negative individuals consume raw and undercooked favorite meat dishes such as "Kitfo" and "kourt" of the Ethiopians. Pork is not consumed at all in the study area.

According to the present findings, no statistically significant difference was observed among different age groups and inhabitants of rural and urban areas of HIV infected and non-infected study participants, and the associated sero-reactivity of anti-*T. gondii* antibodies. The exception to this is the age group 21–30 in the HIV negatives where individuals are 5.58 times more likely to be seropositive for anti-*T. gondii* antibodies. This is in agreement with previous studies on the prevalence of anti-*T. gondii* IgG antibodies from blood donors in Yucatan [33] and seroprevalence of anti-*T. gondii* antibodies in a healthy population from Slovakia [7]. The prevalence of anti-*T. gondii* IgG and IgM antibodies in HIV negative blood donors were 70.29% and 2.97%, respectively. Seroprevalence of IgM that we found was comparable to the 2.4% and 1.9% reported from Czech Republic and Mexico blood donors, respectively [34,35]. The present finding of anti-*T. gondii* IgG antibodies in blood donors is consistent with the 75% prevalence reported from Brazil [36] and 69% from the Southern Mexican State of Yucatan [33]. Consumption of undercooked or raw meat was found to be a risk factor for the presence of anti-*T. gondii* IgG in HIV negative individuals. Undercooked meat consumption has been found to be an important factor in parasite transmission in several studies [18,35]. A similar finding was also demonstrated as risk factor for toxoplasmosis in studies done in Slovakia [7]. Moreover, prevalence of anti-*T. gondii* IgG from male healthy blood donors was significantly higher than female HIV negative blood donors (p=0.001).

In the present study no significant association was found between the seroprevalence of toxoplasmosis and different educational status. Similar findings were recorded between anti *Toxoplasma* IgG antibodies and education level [27] and the infection among pregnant women in Turkey [37].

Generally, IgM antibodies are detectable early after infection and can persist for prolonged times after infection. Therefore, the presence of IgM does not necessarily indicate an acute infection and its presence in subjects with anti *T. gondii* IgG antibodies could indicate a chronic infection [34]. Hence, though we cannot confirm in this study, the finding of sero-positive anti-*T. gondii* IgM/IgG antibodies suggests the potential risk of parasite transmission by blood transfusion practices.

Findings on the association of HIV and toxoplasmosis seroprevalence are varied in different parts of the world. Previous studies involving HIV-infected individuals have reported wide variations in *T. gondii* seroprevalence (3%–22%) [38,39]. Some authors found higher prevalence of *T. gondii* specific IgG in HIV-infected patients compared to non-infected individuals [40,41], whereas others did not find any differences between the two groups [42]. Serologic data provided little information, in extracerebral toxoplasmosis in patients infected with HIV [43]. Previous studies in Ethiopia revealed a higher *Toxoplasma* seroprevalence in both HIV infected and non-infected individuals that is not statistically significantly different between the two groups [29]. On the other hand, studies from Mozambique [44] found similar higher toxoplasmosis occurrence in the HIV-positive groups as compared to HIV-negative groups that according to the authors could be ascribed to common or associated risk factors for both infections, such as exposure to both sexual contacts and meat consumption. Several explanations for the interaction between both infections resulting in high *Toxoplasma* infection in HIV patients have been previously proposed [45]. One possibility is an increase in risky behavior in *Toxoplasma* infected individuals that leads to increased exposure to HIV infection. Such a change in behavior could be due to parasite-driven personality changes, as described in *Toxoplasma*-infected individuals by others [46]. Another plausible explanation is that *Toxoplasma* infection is a marker of exposure to risky social contacts or habits, which correlates with early HIV infection.

Regarding the higher prevalence in males than females, Shimelis et al., [47] observed a statistically significantly higher prevalence rate of *Toxoplasma* infection in males than females in the bivariate analysis, which disappeared in the multivariate analysis. In our case the difference is maintained in the multivariate model and may be attributed to differences in socialization behavior or environmental exposure of males compared to females.

## Conclusion

In conclusion, the present findings showed a high seroprevalence of anti-*T. gondii* antibodies in HIV infected pre-ART and HIV non-infected apparently healthy blood donors in Bahir Dar. Consumption of undercooked or raw meat and contact with cat might greatly contribute towards acquiring *T. gondii* infection. It may be appropriate to include a routine serological screening test for determination anti-*T. gondii* antibodies in HIV infected pre ART individuals and HIV negative apparently healthy blood donors. Those HIV infected individuals, positives for anti-*T. gondii* antibodies should be considered for chemoprophylactic treatment. In addition, health education particularly not to eat undercooked and raw meat, and avoiding contact with cats should be considered.

### Financial support

This study was financially supported by Addis Ababa University and additional funds from Amhara National Regional State Health Bureau.

## Competing interest

The authors declared that they have no competing interest.

## Authors’ contributions

FW conceived the study, undertook statistical analysis and drafted the manuscript. NK, AT and TK initiated the study and made major contributions to the study design and statistical analysis. All authors contributed to the writing of the manuscript and approved the submitted version of the manuscript.
